# Vaccination of newborn monkeys with an influenza HA stem nanoparticle adjuvanted with R848 and AddaVax results in broadened HA recognition and increased antibody secreting cells to HA stem following H1N1 challenge

**DOI:** 10.3389/fimmu.2026.1934178

**Published:** 2026-08-31

**Authors:** Kali F. Crofts, Beth C. Holbrook, Colby J. Hendrix, Ava C. DiPaolo, Courtney L. Page, Rebecca A. Gillespie, Heather A. Burkart, Masaru Kanekiyo, Martha A. Alexander-Miller

**Affiliations:** 1 Department of Microbiology and Immunology, Wake Forest University School of Medicine, Winston-Salem, NC, United States; 2 Vaccine Research Center, National Institute of Allergy and Infectious Diseases, National Institutes of Health, Bethesda, MD, United States; 3 Department of Pathology, Wake Forest University School of Medicine, Winston-Salem, NC, United States

**Keywords:** adjuvants, antibody secreting cell, influenza HA stem, influenza vaccine, memory B cell, newborn, recall response, universal vaccine

## Abstract

**Background:**

How infection shapes recall responses in the setting of preexisting influenza virus hemagglutinin (HA) stem-focused immunity has important implications given the known impact of imprinting on future immune responses. Adoption of an HA-based universal stem vaccine approach for young infants would likely result in stem-specific imprinting. Understanding how imprinting programs memory B cell recall and early antibody responses to HA stem and head in newborns is therefore essential.

**Methods:**

African green monkeys vaccinated with a A/New Caledonia/20/1999 (NC99) hemagglutinin stem-bearing nanoparticle (H1ssF) adjuvanted with R848+AddaVax as newborns were challenged with H1N1 A/California/07/2009 (Ca09). Antibody and draining lymph node cellular responses were analyzed on d7 following challenge. Both quantitative and qualitative aspects of the antibody response were assessed.

**Results:**

Our previous studies showed newborn African green monkeys vaccinated with an R848+AddaVax dual adjuvanted H1ssF nanoparticle exhibited a robust stem-specific antibody response that had broad reactivity and enhanced functional activity. In the present study, we investigated how the response elicited by this vaccine was recalled following infection, evaluating both antibody and cellular responses. While challenge resulted in increases in recognition of many heterologous HA molecules, not all responses showed evidence of boosting at this timepoint. Control (Ctrl) animals had limited neutralizing activity to Ca09 at d7 following infection, whereas challenge resulted in significant increases in neutralizing antibody to the challenge virus. Compared to Ctrl animals, vaccinated infants exhibited lower germinal center B cell responses and IFNγ-producing T follicular helper (Tfh) responses. However, vaccinated infants exhibited a significant increase in HA stem-specific plasmablasts and plasma cells in the lung-draining tracheobronchial lymph nodes together with similar head-specific responses.

**Conclusion:**

Collectively, these findings are consistent with efficient recall of stem-specific responses generated by vaccination with dual adjuvanted H1ssF administered to newborn African green monkeys in response to influenza virus challenge. Interestingly, challenge with the heterologous virus appears to reshape the reactivity profile of the stem-specific response. Further, vaccinated animals exhibit robust early antibody-secreting cell responses, including increased numbers of stem-specific cells, without an apparent reduction in the generation of head-specific cells.

## 1 Introduction

During infancy, there is a heightened vulnerability to respiratory pathogens as the immune system undergoes development (1). Infants under six months of age are particularly susceptible to severe disease following influenza A virus (IAV) infection, with significantly higher rates of influenza-associated hospitalizations (2, 3). The increased vulnerability is exacerbated by the absence of a licensed influenza vaccine in this age group. Together, these factors underscore the urgency of developing effective vaccine strategies that can provide protection against influenza virus infection during the period of infancy.

Newborns have distinct immune alterations that can limit both the generation and durability of certain immune responses. These include diminished antigen-presenting cell (APC) function, reduced proinflammatory cytokine production, increased T regulatory (Treg) cells, and a TH2-skewed CD4 T cell compartment [reviewed in (1)]. Germinal center responses, which are important for the generation of high avidity antibodies, are also constrained (4–7). Despite these alterations, several vaccines demonstrate effective neonatal immunity is achievable. Live-attenuated vaccines such as the Bacillus Calmette-Guérin (BCG) (8–11) and oral polio (12–14) as well as adjuvanted vaccines such as hepatitis B (15, 16), can elicit robust immune responses in young infants. Further, a number of experimental adjuvants are being explored in newborn animal models, including mmCT (detoxified cholera toxin) (17), dmLT (detoxified *E. coli* heat-labile enterotoxin) (17), and the C-type lectin receptor Mincle agonist TDB (trehalose 6,6′-dibehenate) (18). In addition, a growing body of literature, including our own studies, has shown TLR7/8 agonists to be highly effective adjuvants for modulating the response in newborns (19–23). R848 signaling through TLR7/8 promotes proinflammatory responses from DCs and T cells, as well as activation of B cells (22, 24–28). Combination adjuvants are also an area of active research (29). Recently we reported the ability of a dual adjuvanted vaccine (TLR7/8 agonist R848 together with the squalene-based oil-in-water nanoemulsion AddaVax) to induce potent responses in newborn African green monkeys (30). These responses were significantly improved compared to the vaccine containing either adjuvant alone (30).

In addition to the challenges around inducing protective responses, licensed influenza vaccines provide limited protection against drifted or pandemic IAV strains. Antibody responses to both natural infection and seasonal vaccines are predominantly directed to the hemagglutinin (HA) head, the principal site of antigenic drift (31, 32). In contrast, antibodies recognizing the conserved HA stem are more broadly reactive, but typically subdominant (33, 34). To address this, alternative vaccine platforms have been developed to selectively target HA stem-specific antibody generation (35–38). One such platform, the H1ssF nanoparticle vaccine, displays trimeric stem structures on a self-assembling ferritin nanoparticle (37). Vaccination of newborn African green monkeys with a dual adjuvanted (conjugated TLR7/8 agonist resiquimod (R848) plus AddaVax) H1ssF potently stimulated stem-specific antibodies that were both neutralizing and capable of mediating diverse functions, including antibody-dependent cellular phagocytosis (ADCP), antibody-dependent cellular cytotoxicity (ADCC), and antibody-dependent complement deposition (ADCD) (30). This dual adjuvanted approach significantly reduced viral burden in the lungs of vaccinated newborns compared to unvaccinated newborns following IAV H1N1 challenge (30).

Understanding how infection recalls and reshapes immunity after neonatal HA stem-focused vaccination is critical for evaluating stem-based universal influenza vaccine strategies for young infants. Our prior studies showed that newborn African green monkeys vaccinated with H1ssF-R848+AddaVax generated multifunctional HA stem-specific antibodies and exhibited reduced viral burden after H1N1 challenge. However, whether this neonatal stem-focused imprint is efficiently recalled by infection, how it influences early antibody breadth and function, and whether it alters HA stem- versus head-specific B cell responses in the lung-draining lymph node remains unclear. Here, we tested the hypothesis that neonatal H1ssF-R848+AddaVax vaccination establishes stem-focused immune memory that is rapidly recalled after heterologous H1N1 challenge, resulting in early enrichment of stem-specific antibody-secreting cells while preserving the development of head-specific responses induced by infection. To address this, we assessed HA-specific antibody breadth and function together with B cell and Tfh responses in the tracheobronchial lymph node at d7 following Ca09 challenge.

## 2 Materials and methods

### 2.1 Conjugation of R848 to H1ssF-Cys mutant

H1ssF was generated as per (37) and conjugation as in (30). Briefly, the native cysteine at position 31 of *H. pylori* ferritin was substituted to a serine to minimize the chance of disulfide bond formation during biosynthesis. To conjugate R848 to H1ssF, an H1ssF was engineered that replaced a surface accessible serine residue on ferritin with a cysteine (Cys) residue (S111C) based on a previously reported approach (39). The Cys mutant provides an accessible free thiol on the surface of each ferritin subunit for coupling (24 available sites per H1ssF nanoparticle). We linked an R848 amine derivative to the heterobifunctional cross-linker SM(PEG)_4_ (Thermo Fisher Scientific). R848 was added under conditions that maximally saturate the NHS group on SM(PEG)_4._ H1ssF-Cys and R848-SM(PEG)_4_ were incubated for 2hrs at 37^○^C. Non-bound R848-SM(PEG)_4_ was removed by dialysis. H1ssF-R848 was subjected to 3 rounds of ultrafiltration using a 10k Microcon Centrifugal Filter (Millipore) to ensure complete removal of non-bound R848. Vaccine was snap frozen, stored at -80^○^C, and thawed on ice prior to administration.

### 2.2 Study design

Healthy newborn African green monkeys weighing ≥300g were immunized at 3–5 days of age, corresponding to an approximately 12–20 day old human (40). Animals were randomly assigned to different vaccine groups: PBS (n=3) or luciferase mRNA-LNP (n=2) (non-HA vaccinated controls), H1ssF (n=4), or H1ssF-R848+AddaVax (n=7). Animals were assigned to balance sex across groups when possible. 20 μg of H1ssF or H1ssF-R848 together with AddaVax (125 µl of AddaVax (InvivoGen)) was administered intramuscularly into the deltoid muscle of both left and right arms (40 μg of vaccine per dose). A booster dose was similarly administered at d39 or d40pv. On d41 or 45 post boost (pb), newborn African green monkeys were challenged with 1x10^7^ TCID_50_ of A/California/07/09 (Ca09) (BEI Resources, NR-13663). 0.5 mL was delivered by intratracheal installation and 0.125 mL to each nostril. All animals included in the challenge study were followed through d7pc, at which point tissues were collected for analysis. Plasma samples were collected at d1pv, d10pv, d39/40pv, d10/11pb, d41/45pb, and d7pc. Animals administered PBS, luciferase mRNA-LNP, or non-adjuvanted H1ssF were combined to form the Ctrl group for post-challenge analyses because they did not differ significantly in antibody levels following vaccination or challenge. The animal care and use protocol (A23-029) adhered to the US Animal Welfare Act and Regulations and was approved by the Institutional Animal Care and Use Committee. African green monkeys were housed and cared for according to state, federal, and institutional guidelines in facilities accredited by the American Association for Accreditation of Laboratory Animal Care (AAALAC), following the standards established in the Animal Welfare Act and the Guide for the Care and Use of Laboratory Animals.

### 2.3 Detection of HA-stem and total HA immunoglobulin

Trimeric A/NewCaledonia/20/1999 (H1N1) stabilized HA stem protein (37) was used to assess stem-specific antibodies. For HA binding ELISAs, trimeric recombinant full-length HA proteins were used. The panel included: A/New Caledonia/20/1999 (H1) (BEI Resources, NR-48873), A/California/07/2009 (H1) (BEI Resources, NR-42635), A/Singapore/1/1957 (H2) (BEI Resources, NR-52249), A/Wisconsin/67/2005 (H3) (BEI Resources, NR-49237), A/Vietnam/1204/2004 (H5) (VRC, NIH), A/Anhui/1/2013 (H7) (BEI Resources, NR-44365), and A/Hong Kong/33982/2009 (H9) (BEI Resources, NR-41792). We used the FI6V3 HA stem-specific monoclonal antibody as a positive control at 0.156 µg/mL (VRC, NIH) (41). To assess antibody production, ELISA microplates (96-half well) (Greiner bio-one) were coated with 40 ng/well of NC99 trimeric stem or 100 ng/well of recombinant HA protein diluted in PBS (stem ELISAs) or sodium carbonate/bicarbonate coating buffer (pH 9.5) (HA ELISA) overnight at 4 °C. The plates were blocked with 1X blocking buffer (10X Casein Blocking Buffer, Sigma-Aldrich) plus 2% goat serum (Lampire Biologicals) for 1 h and then washed. Wash buffer contained PBS with 0.1% Tween 20. Plasma samples were serially diluted in 1X blocking buffer. HRP-conjugated goat anti-monkey IgM (Fitzgerald #43R-IG074hrp) (1:10,000) and goat anti-monkey IgG (Fitzgerald #43C-CB1603) (1:5,000) were used to detect bound antibodies. Plates were developed using 25 µl of 3,3′,5,5′-Tetramethylbenzidine dihydrochloride (TMB) (Sigma-Aldrich), and stopped after 30 mins by addition of 25 µl of 2N H_2_SO_4_. Plates were read at 450nm on an Elx800 absorbance Microplate Reader (BioTek). For each dilution, the OD_450_ from the no antigen wells was subtracted from the antigen-coated wells. Threshold titer was defined as the value that reached 3X the assay background, i.e., antigen coated wells that did not receive plasma. The values used included plasma dilutions and absorbance 450nm.

### 2.4 Microneutralization assay

The generation of replication-restricted reporter (R3ΔPB1) viruses for H1N1 subtypes (A/California/07/09 & A/New Caledonia/20/99) as well as the rewired R3ΔPB1 (R4ΔPB1) virus for H5N1 (A/Vietnam/1203/04) has been previously described (42). In brief, the R3/R4ΔPB1 viruses were generated by replacing the viral genomic RNA encoding the functional PB1 protein with a gene encoding the fluorescent protein TdKatushka2. These viruses were then rescued through reverse genetics and propagated in the complementary cell line that constitutively expresses PB1. Each R3/R4ΔPB1 virus stock was titrated by measuring the fluorescent units per mL (FU/mL) prior to experimental use. For titration, serial dilutions of the virus stock in OptiMEM were mixed with pre-washed MDCK-SIAT1-PB1 cells (8x10^5^ cells/ml) and incubated in a 384-well plate in quadruplicate (25 µL/well). Plates were incubated at 37 °C with 5% CO_2_ humidified atmosphere for 18–26 h. Following incubation, fluorescent cells were imaged and counted using a Celigo Image Cytometer (Nexcelom) with a customized red filter to detect TdKatushka2 fluorescence. For the microneutralization assay, serial dilutions of Receptor Destroying Enzyme (RDE)-treated plasma were prepared in OptiMEM and mixed with an equal volume of R3/R4ΔPB1 virus (~8x10^4^ FU/mL) in OptiMEM. After 1h of incubation at 37 °C and 5% CO_2_ humidified atmosphere, pre-washed MDCK-SIAT1-PB1 cells (8 x 10^5^ cells/well) were added to the plasma-virus mixtures and transferred to 384-well plates in quadruplicate (25 µL/well). Plates were incubated and counted as previously described. The target virus control range for this assay was 500 to 2,000 FU per well, with the cell-only control being acceptable up to 30 FU per well. Percent neutralization for each well was calculated by setting the virus control (virus plus cells) as 0% neutralization and the cell-only control (no virus) as 100% neutralization. A 7-point neutralization curve was plotted against serum dilution for each sample, and the 50% inhibitory concentration (IC_50_) was calculated using a four-parameter nonlinear fit generated in Prism (GraphPad).

### 2.5 Antibody-dependent cellular cytotoxicity

KHYG-1 cells transduced with rhesus macaque CD16 (43) were used to conduct the ADCC assay, adapted from (44). ELISA plates (Corning) were coated with 50 µL (2 µg/mL in PBS) of recombinant Ca09 HA protein (BEI Resources) and incubated overnight at 4 °C. The plates were then washed and blocked with PBS containing 5% bovine serum albumin (BSA) at 37 °C in a 5% CO_2_ incubator for 30 mins. After removing the blocking solution, heat inactivated plasma samples were serially diluted (starting dilution 1:10) and incubated at 37 °C in a 5% CO_2_ incubator for 2 hrs. Following additional washes, 1x10^5^ cells KHYG-1 MmCD16 cells were added per well (50 µL) in media (RPMI + 10% FBS + 1% L-glut, 0.2% Primocin) with recombinant human IL-2 (Biolegend, #589104) (10 U/mL, 50 µg/mL). CD107a PE (Biolegend, #328608) (1:50) was diluted into 25 µL of warmed media and then added to cells. Cells were cultured in the presence of Golgi Plug (1:1000) and Golgi Stop (1:1500) (BD Biosciences) for 5 h at 37 °C in a 5% CO_2_ incubator. After incubation, cells were transferred to a 96 V-bottom plate, washed with PBS and stained with Zombie Violet (Biolegend) for 15 mins at room temperature (RT) in the dark. Following washes with PBS + 1% FBS, cells were fixed with 2% paraformaldehyde. ADCC activity was assessed using flow cytometry on a BD X20 Fortessa cytometer. The percentage of CD107a+ NK cells was calculated by subtracting the PBS control values from the HA specific values for each dilution.

### 2.6 Antibody-dependent cellular phagocytosis

THP-1 cell phagocytosis of HA coated beads was performed as previously detailed (45). Biotinylated Ca09 HA (kindly provided by the DCVC, Duke University) was adsorbed onto 1.0 µm of fluorescently labelled beads called FluoSpheres™ NeutrAvidin™ (yellow-green fluorescent (505/515)) at a ratio of 10 µg protein to 10 µl beads and incubated overnight at 4 °C. In a 96-round bottom plate, 10 µl of HA coated beads were then incubated with 10 µl of heat-inactivated plasma diluted in PBS (2-fold dilutions, starting dilution 1:800) for 2 h at 37 °C. After washing away any unbound antibody, THP-1 cells (RPMI + 10% FBS + 1% L-glut, 1% P/S) (5x10^4^ cells/well) were added and incubated for 16 h at 37 °C. Following incubation, the cells were fixed, and phagocytosis was analyzed using flow cytometry on a BD X20 Fortessa (BD Biosciences). Phagocytosis scores were calculated by multiplying the percentage of bead positive cells by the geometric mean fluorescence intensity and dividing by 1000 at the 1:800 dilution.

### 2.7 Antibody-dependent complement deposition

Antibody-dependent complement deposition of Ca09 HA coated beads was performed as previously outlined (46). HA coated 1 μm non-fluorescent NeutrAvidin™ beads were prepared as in the ADCP assay and incubated with serially diluted, heat-inactivated plasma samples (2-fold dilutions, starting dilution 1:5) for 2 hours at 37 °C. Lyophilized guinea pig complement (Cedarlane, CL4051) was reconstituted in ice-cold dH_2_0, then diluted in 1:60 veronal buffer with 0.1% gelatin (GVB ++. Boston BioProducts, #IBB-300X). Following this, 150 μl of the diluted complement was added to the opsonized beads and incubated for 20 minutes at 37 °C. The beads were subsequently washed with 15 mM EDTA and stained with anti-guinea pig C3-FITC (MP Biomedicals, # 855385). After washing, ADCD was analyzed by flow cytometry using a BD X20 Fortessa (BD Biosciences). Complement scores were calculated by multiplying the percentage of C3+ beads by the GMFI and dividing by 1000, using the 1:5 dilution.

### 2.8 Detection of HA head and stem-specific B cells and antibody secreting cells

Flow cytometry analysis of the B cell response following challenge was performed as previously described (47). TBLN cells were thawed and 1x10^6^ cells were plated in 96-well U-bottom plates for staining. Cells were pelleted and stained with Zombie Aqua fixable viability dye (Biolegend) for dead cell discrimination. To minimize non-specific binding, 2.5 ug of Fc Block (BD Biosciences, 564220) was applied. For identification of HA-head and HA-stem specific B cell populations, cells were labelled with fluorescent HA Ca09 (H1) and IN05 (H5) probes, provided by the NIH VRC. Cells positive for both probes (H1^+^H5^+^) were classified as HA stem-specific, whereas those that bound only H1 probe (H1^+^H5^-^) were considered HA head-specific.

For surface antibody characterization, the cells were stained with BUV395 anti-IgM (clone G20-127; BD Biosciences, 563903), BV510 anti-IgG (clone G18-145; BD Biosciences, 563247), FITC anti-CD38 (clone AT-1; STEMCELL Technologies, 100-1578), PeVIO770 anti-CD3 (clone 10D12; Miltenyi Biotec, 130-116-628), AF700 anti-CD20 (clone 2H7; BD Biosciences, 560631), V450 anti-CD45 (clone D058-1283; BD Biosciences, 561291), BV786 anti-CD11b (clone M1/70; BD Biosciences, 740861), along with the PE-conjugated H1 and APC-conjugated H5 probes (NIH VRC). Brilliant Stain Buffer (BD Biosciences, 563794) was used as recommended by the vendor. Cells were permeabilized for 1 hour at ambient temperature in the dark using the FoxP3/Transcription Factor Buffer Set (eBioscience, 00-5523). Intracellular labeling was performed to evaluate Ki-67 and BCL-6 expression, as well as the binding of HA probes to intracellular IgM/IgG. The antibody panel for intracellular staining consisted of BUV395 anti-IgM (clone G20-127; BD Biosciences, 563903), BV510 anti-IgG (clone G18-145; BD Biosciences, 563247), BUV737 anti-Ki-67 (clone B56; BD Biosciences, 567130), PerCpCy5.5 anti-BCL-6 (clone K112-91; BD Biosciences, 562198), together with PE-labeled H1 and APC-labeled H5 probes (NIH VRC). Data acquisition was performed on a BD LSR Fortessa X-20 flow cytometer (BD Biosciences), and analyses conducted using BD FACSDiva software (BD Biosciences).

### 2.9 Detection of Ca09 HA and NC99 stem specific T cell responses

Flow cytometry analysis of the T cell response following challenge was performed as previously described (47). TBLNs were thawed and plated at 1x10^6^ cells per well in 96-well flat-bottom plates using RPMI-1640 medium supplemented with 2 mM L-glutamine (Gibco), 0.1 mM sodium pyruvate (Gibco), 100 U/mL penicillin (Gibco), nonessential amino acids (1X) (Gibco), 100 µg/mL of streptomycin (Gibco), 10 mM HEPES (Gibco), 0.05 mM 2-mercaptoethanol (Sigma-Aldrich), and 10% fetal bovine serum (Atlanta Biologics). Cells were stimulated with either HA-stem or Ca09 HA peptide pools. HA stem peptide pools were generated from influenza A/New Caledonia/20/1999 (H1N1) HA proteins (BEI Resources, NR-2602), comprising peptides 2-10 (D18-L58) and 50-90 (A292-I529). Each peptide was 16–17 amino acids long with 11–12 amino acid overlaps. Peptides were dissolved in DMSO (Sigma-Aldrich, D2650) (final well concentration of 0.2% DMSO). For Ca09 HA stimulation, PepMix from influenza A (HA/Cal/H1N1) (JPT Peptide Technologies, 56080) was used, also reconstituted in DMSO (0.2% final). The final concentration of each peptide was 0.5 µg/mL of NC99 HA-stem and 1 µg/mL for Ca09 HA PepMix. Cells were stimulated with peptide pools for 14 hours at 37 °C, in 5% CO_2_ incubator. Intracellular cytokine production was measured after 14 hours of stimulation. Brefeldin A (1:1000; GolgiPlug, BD Biosciences) and monensin (1:1500; GolgiStop, BD Biosciences) was added 3hours after initiation of the stimulation period.

### 2.10 ELISpot analysis for detection of antibody secreting cells in the bone marrow

Millipore Multiscreen 96 well plates (MSIPS4W10) were pre-wet with 35% EtOH and washed with sterile dH_2_O. Wells were coated overnight at 4 °C with 40ng/well of Ca09 stabilized trimeric stem in DPBS. Control wells received DPBS. Plates were washed with DPBS and blocked with RPMI 1640 + 10% FBS at room temperature for a minimum of 30 minutes. Bone marrow cells (1x10^6^/well) were added in triplicate and incubated at 37 °C for 19 hours. Plates were washed with DPBS and goat anti-monkey IgG-HRP (Fitzgerald 43C-CB1603) in DPBS + 0.5% FBS was added. After washing TMB Substrate Solution (Mabtech 3651-10) was added to wells and developed until spots were visible (5–15 minutes). Plates were imaged and quantified using CTL S6 Ultra-V Analyzer (ImmunoSpot 7.0.38.14). Spots were calculated by averaging triplicate wells.

### 2.11 Statistical analysis

Multiple analytic methods were employed in these studies. The statistical details of each experiment can be found in the figure legends. For analyses assessing the effects of different vaccine groups over time, two-way repeated measures ANOVA models were utilized. If there was evidence of overall treatment effects, then group comparisons at each time point were made were made, adjusting for multiple comparisons using the Holm-Šídák test. For analyses comparing vaccine groups at a single time point, one-way ANOVA models were applied. When overall treatment effects were observed, pairwise comparisons were conducted using either Holm-Šídák method to account for multiple comparisons. Correlations were calculated and analyzed for associations between two continuous variables. For all models, p values were defined as *p=≤ 0.05, **p ≤ 0.005, ***p ≤ 0.001, ****p ≤ 0.0001. Analyses were performed using Prism 10.6.1 (GraphPad, La Jolla, CA, USA).

## 3 Results

### 3.1 Challenge of African green monkeys vaccinated with H1ssF-R848+AddaVax as newborns boosts reactivity for many but not all HA subtypes

**Figure 1:**
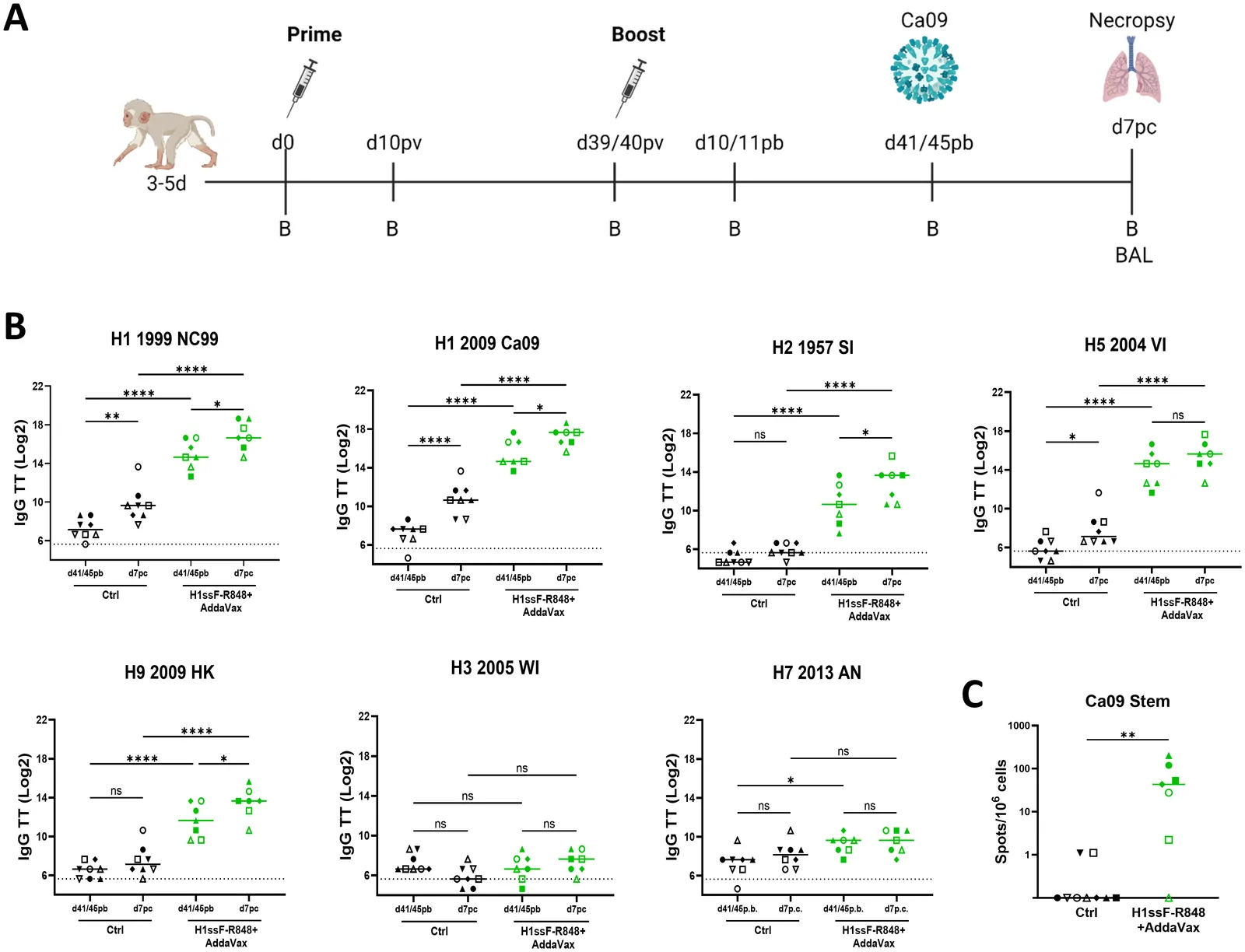
HA reactivity of antibodies present at d7pc in Ctrl and H1ssF-R848+AddaVax vaccinated animals. Newborn African green monkey vaccination and sampling schedule **(A)**. IgG binding to group 1 and group 2 HA molecules in plasma at d41/45pb or d7pc **(B)**. The dotted line indicates the LOD for the assay. Stem-specific IgG secreting antibody secreting cells in the bone marrow at d7pc **(C)**. Threshold titer (TT) was defined as the highest dilution with an OD450 greater than 3X assay background. Experimental groups included: Ctrl (n=9) and H1ssF-R848+AddaVax (n=7). Each animal is represented by a distinct symbol used consistently throughout the study. Lines represent the median. Statistical significance was determined using a one-way ANOVA **(B)** with Holm-Šídák’s multiple comparisons test, or Mann-Whitney test **(C)**. Not significant ns, *p=≤0.05, **p=≤0.01, ****p=≤0.0001. Image in **(A)** created with BioRender. D41/45pb data were previously published (30) and are shown here to assess the response to challenge.

We first asked whether infection recalled or further broadened the HA-reactive antibody response elicited by neonatal H1ssF-R848+AddaVax vaccination. HA-specific IgG antibody was quantified in the plasma of control (Ctrl) or H1ssF-R848+AddaVax vaccinated newborn animals on d7 following challenge with Ca09 virus (**Figure 1A**). The Ctrl group consisted of animals that received PBS, a luciferase mRNA-LNP, or non-adjuvanted H1ssF. Our previous analysis of the response elicited by non-adjuvanted H1ssF showed this platform was not immunogenic in the absence of adjuvant, i.e., antibody responses to stem were not detected (30, 48). Given the absence of a significant difference in the stem-specific antibody levels across the PBS, luciferase mRNA-LNP, and H1ssF groups following either vaccination or challenge, these animals were combined to increase the power of our analysis and are hereafter referred to as the control (Ctrl) group.

Antibodies elicited by H1ssF-R848+AddaVax were capable of broad recognition of Group 1 HAs (d41/45p.b., **Figure 1B**). By d7pc, there was a significant increase in recognition of H1 (NC99 4.0-fold, Ca09 3.3-fold), H2 (4.4-fold), and H9 (3.6-fold), but interestingly not H5 (**Figure 1B**). In the Ctrl group significant recognition was observed for Ca09 (H1), NC99 (H5), and VI (H5) molecules. Together, these data indicate that Ca09 challenge of vaccinated animals was associated with increased recognition of several, but not all, heterologous HA molecules at d7pc. Thus, rather than uniformly boosting all stem-reactive specificities, infection appeared to alter the detectable HA reactivity profile of the vaccine-induced response.

### 3.2 Stem-specific IgG antibody secreting cells are present in the bone marrow of H1ssF-R848+AddaVax vaccinated animals

The presence of HA stem-specific antibodies detected in circulation at the time of challenge along with the evidence of a recalled antibody response suggested vaccination resulted in long lived antibody secreting cell responses. To explore this, we assessed stem-specific IgG secreting cells in the bone marrow on d7pc using the sensitive ELISpot approach. We reasoned that this early timepoint would primarily reflect vaccine-induced seeding of antibody secreting cells in the bone marrow, given previous reports of the kinetics of this process following respiratory virus infection (49, 50). In support of this we saw no appreciable response in Ctrl animals (**Figure 1C**). In contrast, all but one animal in the H1ssF-R848+AddaVax vaccinated group had detectable stem-specific IgG secreting cells (**Figure 1C**). These cells were likely generated by the original vaccination regimen, although we cannot rule out the possibility that some infection recalled memory B cells underwent rapid differentiation into antibody secreting cells and trafficked to the bone marrow at this early timepoint.

### 3.3 H1ssF-R848+AddaVax vaccinated animals have higher neutralizing antibodies to the Ca09 challenge virus at d7pc

**Figure 2:**
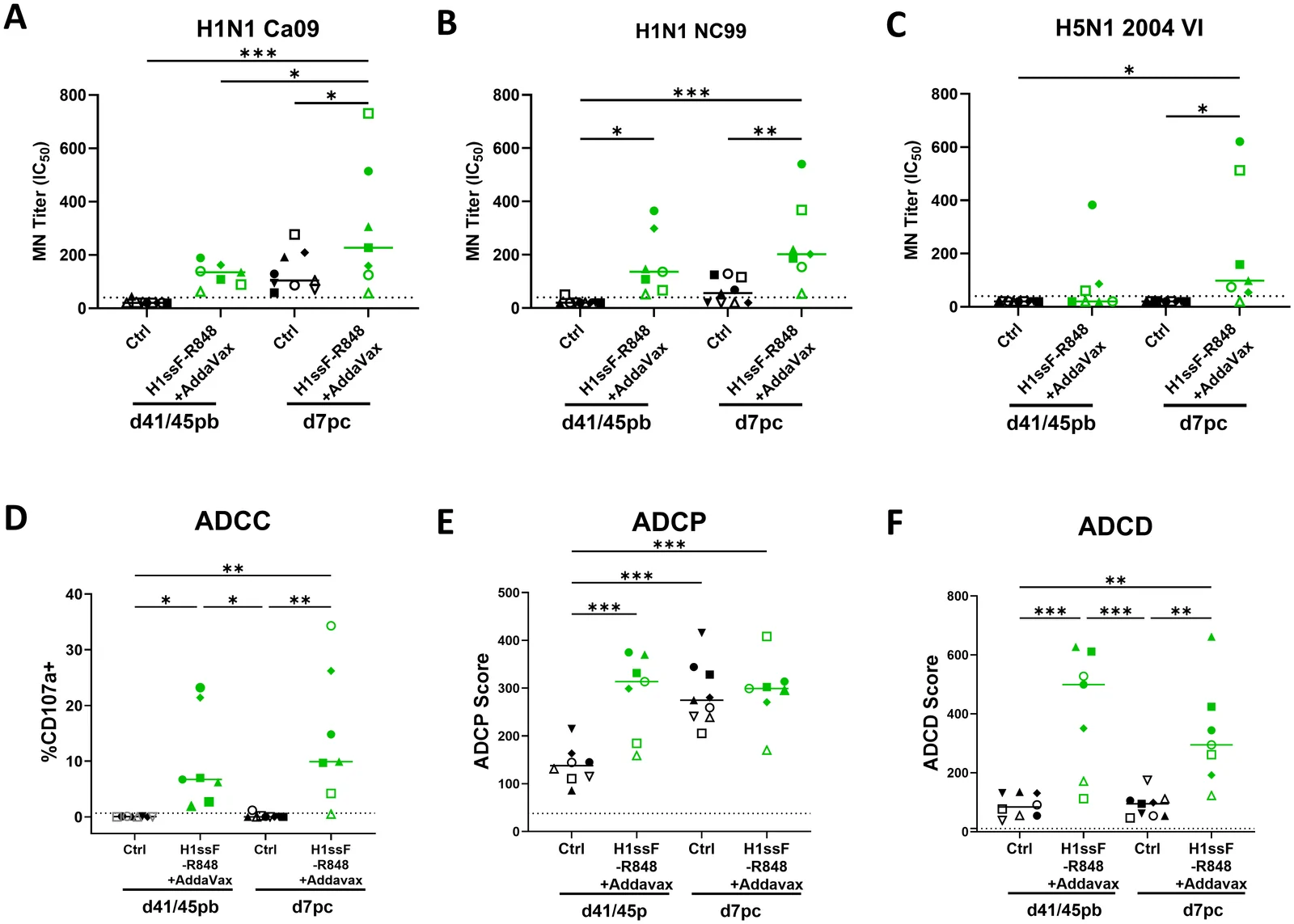
H1ssF-R848+AddaVax vaccination elicited antibody responses with greater neutralizing potential and enhanced Fc effector activities at d7pc compared to Ctrl animals. Plasma samples from newborn African green monkeys on d7pc were assessed for neutralizing **(A–C)** and Fc effector activity **(D–F)**. Responses at d41/45pb are shown to assess challenge induced changes. Neutralizing IC_50_ titers against H1N1 A/New Caledonia/20/99 **(A)**, H1N1 A/California/07/09 **(B)**, and H5N1 A/Vietnam/1203/04 **(C)** were evaluated using a reporter-based microneutralization assay. ADCC activity **(D)** was determined by flow cytometry as the percentage of CD107a+ KHYG-1 cells (1:10 dilution of plasma). ADCP scores **(E)** were quantified as bead positive THP-1 cells×GMFI/1000 (1:3200 dilution of plasma). ADCD scores **(F)** were determined by calculating the percentage of C3+ beads×GMFI/1000 (1:5 dilution of plasma). The dotted line represents the LOD for the assay. Lines represent the median. Statistical significance was determined using a one-way ANOVA with a Holm-Šídák’s multiple comparison test. Not significant (ns), *p=≤0.05, **p=≤0.01, ***p=≤0.001. D41/45pb data were previously published (30) and are shown here to assess the response to challenge.

We next determined whether the early post-challenge antibody response differed across functional axes, including neutralization and Fc-mediated effector activity given the increasing evidence that non-neutralizing antibody functions contribute significantly to the control of influenza virus following infection (e.g (51, 52).,). Neutralizing activity was assessed using a panel of replication-restricted reporter influenza viruses that included Ca09 (H1N1), NC99 (H1N1), and A/Vietnam/1203/2004 (2004 VI) (H5N1) (**Figures 2A–C**) (42). By d7pc, there was a significant increase in neutralizing activity against Ca09 H1N1 (**Figure 2A**). Head-specific antibodies likely contribute to the increase in neutralizing activity against Ca09 given the significant increase observed in Ctrl animals; however, this contribution seems unlikely to solely account for the high levels obtained in many of the vaccinated infants. Neutralizing antibodies to NC99 and H5N1 were also elevated in vaccinated animals as a result of challenge, although this did not reach statistical significance (**Figures 2B, C**). How these levels may evolve at later timepoints remains an open question. Nonetheless, the levels of NC99 and H5N1 neutralizing antibody at d7pc were significantly higher than in Ctrl animals, where neutralizing activity was minimal or absent (**Figure 2C**).

We next evaluated the role of vaccination on non-neutralizing functional attributes (ADCC, ADCP, and ADCD activity) of the antibodies present following challenge. ADCC activity was measured via CD107 upregulation on KHYG-1 cells transduced with rhesus macaque CD16 (43). ADCP activity was assessed through internalization of fluorescently labeled HA-coated beads and ADCD by C3 deposition on HA-coated beads. Interestingly, viral challenge did not significantly increase any of these activities in the H1ssF-R848+AddaVax newborns within the 7 day challenge period assessed (**Figures 2D–F**). This may reflect the early timepoint assessed. Ctrl newborns exhibited increases only in ADCP. This activity was rapidly elicited following infection and surprisingly, had reached a level similar to that found in H1ssF-R848+AddaVax vaccinated newborns at d7pc (**Figure 2E**).

Overall, the d7pc antibody profile suggests that vaccination impacted the neutralizing antibody axis during the early recall response, particularly against the Ca09 challenge virus. In contrast, ADCC, ADCP, and ADCD activities were not significantly increased by challenge in vaccinated animals over this same interval. Finally, ADCP activity in Ctrl animals reached levels comparable to vaccinated animals by d7pc. These data indicate that early infection-induced recall after neonatal H1ssF-R848+AddaVax vaccination does not enhance all antibody functions equivalently and may differentially shape neutralizing and Fc-mediated effector activities during the early response. However, we note that additional regulation of the response may occur at later times following infection as the response evolves.

### 3.4 H1ssF-R848+AddaVax vaccinated newborns have limited evidence of GC responses at d7pc compared to Ctrl animals

We next explored how the presence of vaccine-induced stem-focused immunity altered early HA-specific GC B cell and Tfh responses in the lung-draining TBLN. Early following challenge, memory B cells can enter newly formed GCs (53), with higher affinity memory B cells efficiently differentiating into antibody secreting cells (54, 55). Naïve B cells can also be recruited into GCs to promote diversification of the response, a process thought to provide protection against drifted viral epitopes (55).

**Figure 3:**
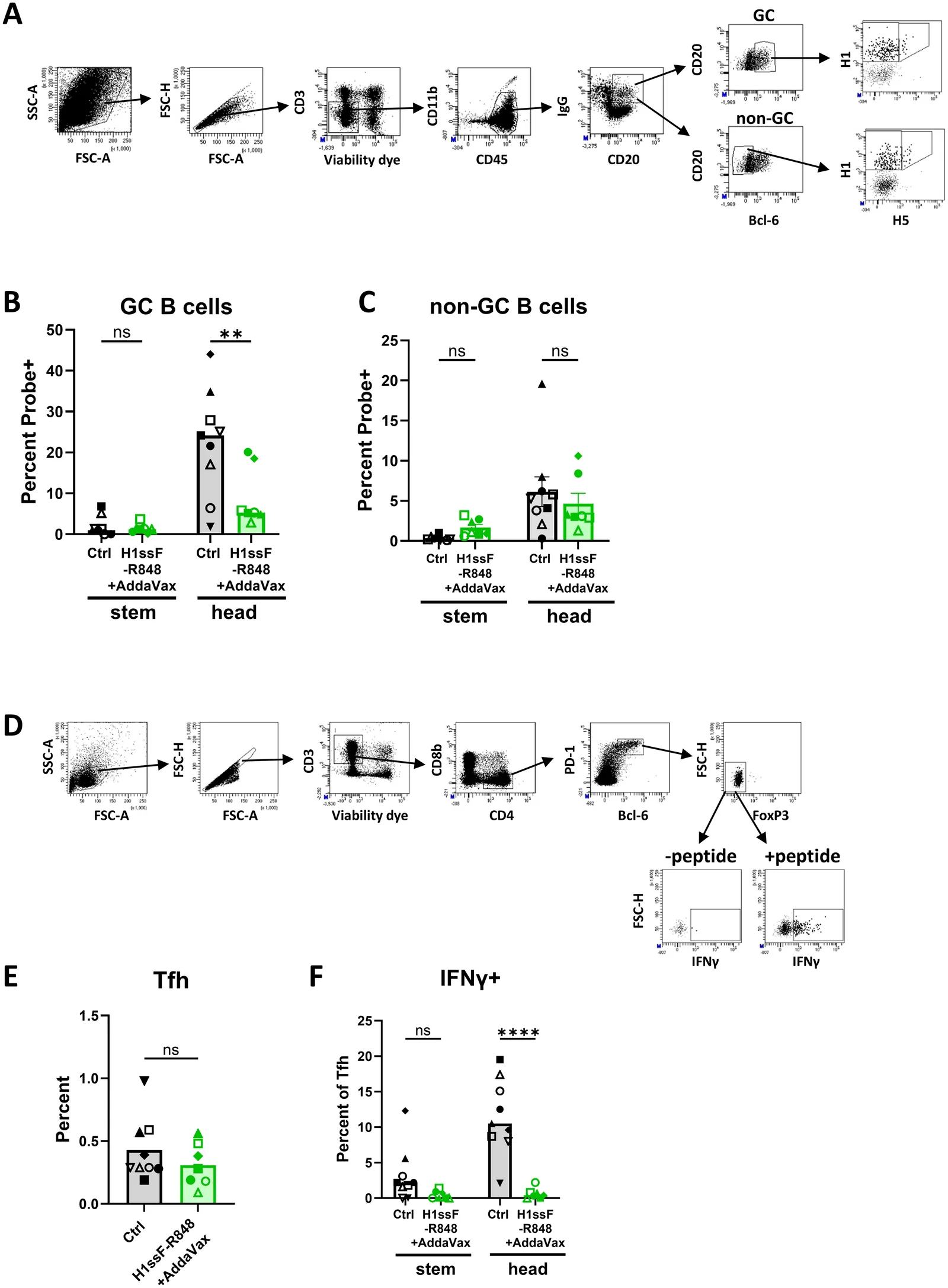
The H1ssF-R848+AddaVax-induced response results in reduced HA-specific GC B cell and IFNγ-producing Tfh responses at d7pc. Gating strategy to identify HA head (H1^+^H5^-^) and HA stem (H1^+^H5^+^)-specific GC B cells (CD20^+^IgG^+^BCL-6^+^) and non-GC B cells (CD20^+^IgG^+^BCL-6^-^) in the TBLN at d7pc **(A)**. Percentage of HA head-specific and stem-specific B cells within the GC **(B)** or non-GC **(C)** B cell population. Gating strategy to identify Tfh cells (CD3^+^CD4^+^PD-1^hi^BCL-6^+^FoxP3^-^ cells) **(D)**. Percentage of Tfh cells within the CD3^+^CD4^+^ population **(E)**. Percentage of IFNγ^+^ cells within the Tfh cell population following stimulation with Ca09 HA or NC99 stem peptide pools **(G)**. Data represent the median. Statistical significance was determined using a two-way ANOVA with a Holm-Šídák’s multiple comparison test **(B–D)** or unpaired Student’s t test **(E)**. Not significant (ns), **p=≤0.05, ****p=≤0.0001.

Head and stem-specific B cells in the TBLN were quantified on d7pc using fluorescently labeled Ca09 HA (H1) and A/Indonesia/05/2005 (IN05) (H5) probes (56). Co-staining with the probes identifies HA stem-specific B cells (H1^+^H5^+^), whereas HA head-specific B cells are positive only for the H1 probe (H1^+^H5^-^) (57) (gating strategy and representative data shown in **Figure 3A**). In the H1ssF-R848+AddaVax vaccinated newborns, we expect the stem-specific response to be comprised predominantly of recalled memory B cells at d7pc, while the head-specific response reflects naïve cells recruited by infection.

To determine how vaccination with H1ssF-R848+AddaVax impacted the differentiation of responding B cells, we quantified cells with a GC phenotype (IgG^+^CD20^+^BCL-6^+^). BCL-6 is the master transcriptional regulator of GC B cells and is essential for GC B cell development and maintenance (58, 59) Surprisingly, no significant difference in the percentage of stem-specific cells within the GC B cell population was found in vaccinated versus Ctrl animals (**Figure 3B**). In contrast, the percentage of head-specific B cells within the GC population was significantly elevated in Ctrl newborns (4.6-fold increase) (**Figure 3B**). No significant differences were observed with vaccination in the frequencies of these populations within non-GC B cells (**Figure 3C**).

Tfh cells are important regulators of the GC response, providing critical help to GC B cells through the secretion of cytokines and the expression of CD40L (60). Further, they have been reported to play a critical role in class switching and memory B cell accumulation in the lung following influenza virus infection (61, 62). Thus, we sought to understand whether the differences in GC responses were associated with the development of Tfh cells (CD3^+^CD4^+^PD-1^hi^BCL-6^+^FoxP3^-^, gating strategy shown in **Figure 3D**). Tfh cells in the lung draining TBLN from vaccinated or Ctrl animals had similar representation of this subset (**Figure 3E**). To probe the presence of HA-specific cells, we evaluated antigen-induced IFNγ production. We chose this approach due to the challenges in detecting IL-21 by flow cytometry in African green monkeys and the knowledge that IFNγ-producing Tfh cells are associated with induction of effective influenza virus-specific antibody responses (62). TBLN cells were stimulated with Ca09 HA or NC99 stem peptide pools and IFNγ production in Tfh cells quantified. In agreement with the increase in head-specific GC B cells, on average Ctrl animals exhibited a significantly increased (26.3-fold) percentage of IFNγ^+^ cells within the Tfh population following stimulation with the Ca09 HA (**Figure 3F**). Appreciable IFNγ was not detected in Tfh cells from vaccinated animals. Together, these data suggest that, at d7pc, vaccinated animals had reduced evidence of HA-specific GC B cell and IFNγ-producing Tfh responses compared with infected Ctrl controls. Because this analysis was limited to a single early time point, additional studies are needed to determine whether vaccination delays, reduces, or alters the kinetics of GC responses following infection.

### 3.5 H1ssF-R848+AddaVax vaccinated newborns have higher stem-specific plasmablasts and plasma cells at d7pc compared to Ctrl animals

**Figure 4:**
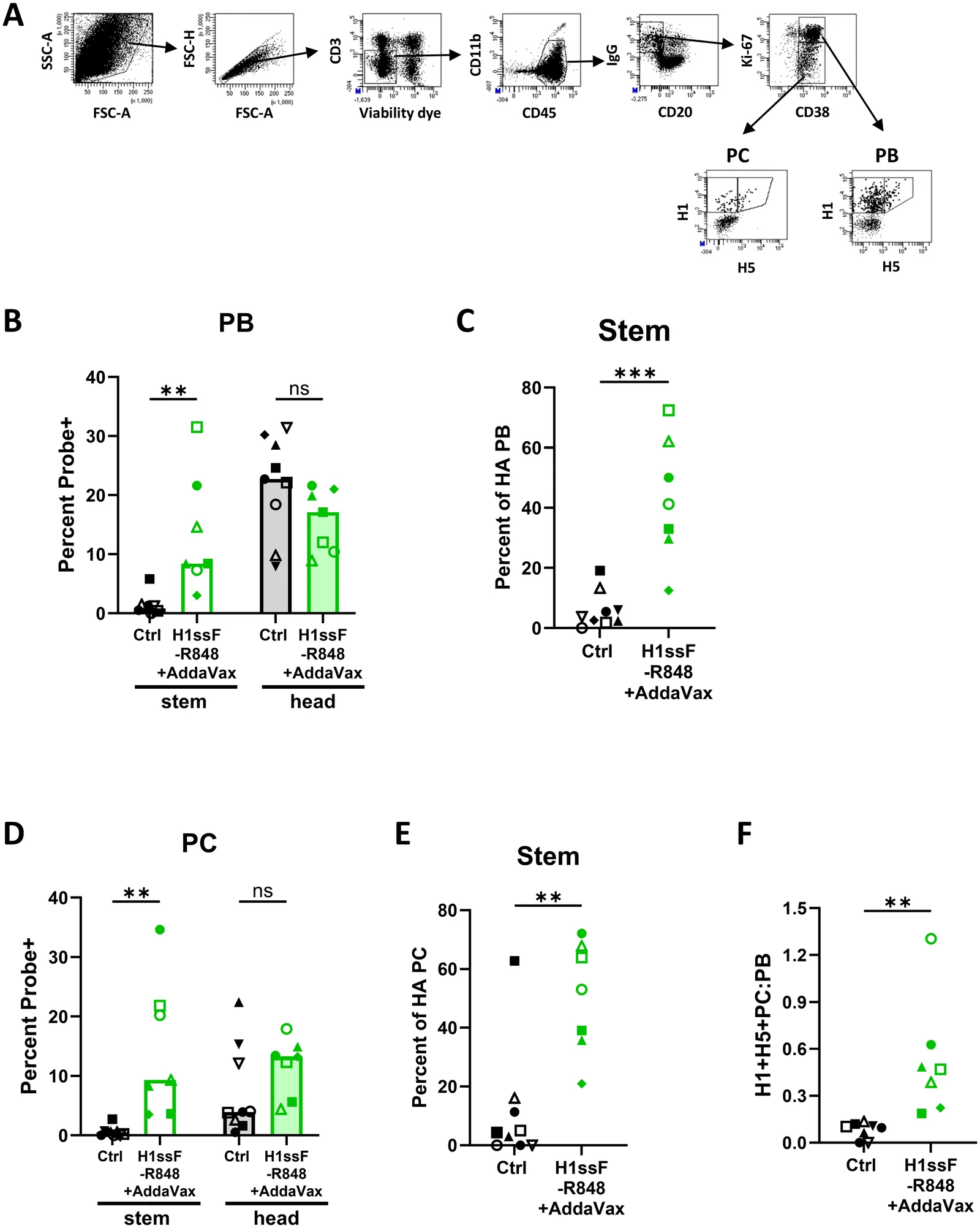
H1ssF-R848+AddaVax vaccinated newborns exhibit significant increases in the HA stem-specific plasmablast and plasma cell responses in the TBLN following challenge. Gating strategy to identify PBs (CD20^-^IgG^+^CD38^+^Ki-67^+^) and PCs (CD20^-^IgG^+^CD38^+^Ki-67^-^) in the TBLN on d7pc **(A)**. Percentage of HA head (H1^+^H5^-^) and HA stem (H1^+^H5^+^) cells within the PB population **(B)**. Proportion of HA stem^+^ cells within the total H1^+^ PB population **(C)**. Percentage of HA head (H1^+^H5^-^) and HA stem (H1^+^H5^+^) cells within the PC population **(D)**. Proportion of HA stem^+^ cells in the total H1^+^ PC population **(E)**. Ratio of stem-specific PC: PB **(F)**. Bars represent the median. Statistical significance was determined using a two-way ANOVA with a Holm-Šídák’s multiple comparison test **(B, D)** or unpaired Student’s t test (C,E,F). Not significant p>0.05 (ns), **p=≤0.01, ***p=≤0.001.

Finally, we evaluated whether there was evidence of recall and differentiation of vaccine-elicited memory B cells into the antibody-secreting cells in the TBLN at d7pc. We evaluated HA head and stem-specific cells within the PB (IgG^+^CD38^+^Ki-67^+^CD20^-^) and PC (IgG^+^CD38^+^Ki-67^lo/-^CD20^-^) populations (gating strategy shown in **Figure 4A**). Cells were permeabilized prior to probe staining to allow detection of intracellular IgG (63) given the highly reduced expression of membrane-associated BCR in these populations (64). Stem, but not head-specific PB and PC were more highly represented in animals vaccinated with H1ssF-R848+AddaVax as newborns (**Figures 4B, D**). Further, stem-specific cells comprised a higher percentage of the total HA-specific PB and PC populations in H1ssF-R848+AddaVax vaccinated compared to Ctrl animals (**Figures 4C, E**). Finally, the stem-specific antibody secreting cell response was more highly skewed towards PC in the vaccinated compared to Ctrl animals, suggesting efficient rapid maturation into this terminally differentiated state at this early timepoint (**Figure 4F**).

## 4 Discussion

We previously reported the ability of a dual adjuvanted (R848+AddaVax) H1ssF nanoparticle vaccine delivered to newborn African green monkeys to induce a multifunctional (neutralizing, ADCC, ADCP, and ADCD) antibody response that resulted in increased protection following challenge (30). Here, we evaluated how the potent HA stem-restricted memory response elicited by vaccination was recalled and further, how it impacted the early HA-specific B cell and antibody responses generated by viral challenge. This question is of importance because imprinting by initial antigen exposure exerts a strong effect on future immune responses (33, 65), and adoption of an HA-based universal stem vaccine approach administered to influenza virus naïve infants would establish a restricted stem-specific immune background. Five notable findings emerge from our study. First animals vaccinated with H1ssF-R848+AddaVax as newborns had an antibody pool capable of broader HA recognition at d7 following infection compared to animals that were naïve to HA. Second, vaccinated animals had a higher level of Ca09 neutralizing antibodies than Ctrl animals. Third, there was evidence of rapid recall within the HA stem-specific memory pool resulting in increased PC and PB in the lung-draining TBLN at d7pc. Fourth, vaccinated animals showed reduced evidence of HA-specific GC B cell and IFNγ-producing Tfh responses at d7pc compared with infected HA-naïve controls. Fifth, head-specific PB and PC responses were readily detected in vaccinated animals and not significantly different from the Ctrl group. This is an important finding as it suggests early head-specific antibody secreting cell responses are not impaired by the presence of the vaccine-induced stem-specific response.

In contrast to the antibody secreting cell response, we did not find evidence of increased stem-specific GC responses with vaccination. In fact, stem-specific GC B cells were limited in both groups. In contrast, GC B cells specific for head were readily detected in both groups; although they were significantly lower in vaccinated animals. We speculate the limited stem-specific GC cells in vaccinated animals may reflect rapid differentiation of memory B cells into antibody secreting cells. Recruitment of remaining stem-specific memory B cells or naïve cells may be hampered by pre-existing antibody that could limit access of B cells to shared stem epitopes through epitope masking, as has been reported in a mouse model of HA vaccination (66). However, this explanation would not account for the concurrent reduction in head-specific GC cells, which could instead reflect the lower viral load present in vaccinated animals (30). In Ctrl animals, the modest number of stem-specific GC B cells is likely the result of subdominance together with the early timepoint evaluated given reports that subdominant responses may emerge later in the GC response (67). Uncovering the mechanistic basis of our findings and whether they reflect the mature virus-specific response requires further studies.

The limited HA-specific GC B cells in vaccinated animals was coupled with nearly undetectable IFNγ-producing HA-specific Tfh cells at d7pc. We note that our ability to interpret this finding is limited because Tfh responses were not measured over time in this study. Previously, we showed that this vaccine induced circulating IL-21 and IFNγ-producing CD4 T cells (30); however, these responses were modest and whether they persisted until time of challenge is unknown. The limited induction of HA-stem Tfh cells is consistent with previous work by Tan et al., demonstrating that stem-directed influenza immunogens elicit poor Tfh cell responses despite inducing stem-specific antibodies. Additionally, reduced Tfh cell responses may reflect decreased antigen availability and an altered inflammatory environment resulting from lower viral loads in vaccinated animals. We posit Tfh cell help may have been insufficient to support the entry of memory B cells or naïve cells into the GC reaction in vaccinated animals. If indeed the modest Tfh response measured here persists over the course of the response, targeting increased Tfh generation in the context of H1ssF vaccination may be a strategy to promote improved GC responses following challenge. Future studies with longer evaluation times following challenge are needed to address this important question.

We found prior vaccination resulted in a greater breadth of HA reactivity following infection at this timepoint than was present in the Ctrl animals. Increased breadth with vaccination versus infection has been reported in an adult NHP model (68). In that study, adult rhesus macaques were infected with A/Aichi/02/1968 (H3N2) or vaccinated with recombinant HA protein ectodomain from the same strain together with an oil-in-water adjuvant containing R848 and CpG oligonucleotides (68). Antibody responses in infected animals exhibited strong neutralizing activity but had limited breadth. In contrast, vaccination with HA protein resulted in a weaker response with little neutralizing activity, but substantial breadth. We previously reported the pattern of reactivity following challenge differed in the dual adjuvanted animals compared to those administered H1ssF with AddaVax alone (47). We would speculate that this may reflect differences in the clones that were recruited into the response or alternatively, differences in SHM and selection in the GC that is controlled by the adjuvant conditioned environment.

Several limitations should be considered when interpreting these findings. First, cellular analyses in the lung draining TBLN were performed at a single early time point, d7pc, which limits conclusions about the magnitude, durability, and kinetics of GC, Tfh, memory B cell, and antibody secreting cell responses. The reduced GC and Tfh responses observed in vaccinated animals may reflect altered timing rather than a sustained reduction in these compartments. Second, group sizes were relatively small, reflecting the constraints of neonatal nonhuman primate studies, and this may have limited the ability to detect more modest differences across vaccine groups or antibody functions.

In conclusion, the maintenance and boosting of a broadly reactive antibody response in the face of virus challenge will be a critical aspect of employing a stem-based universal influenza vaccine approach. As was observed in recalled responses when AddaVax was included as the sole adjuvant (47), vaccination with the HA stem is associated with beneficial effects on the early generation of stem-specific antibody secreting cells. These data extend our understanding of the early recall response generated following challenge of NHP vaccinated with H1ssF-R848+AddaVax as newborns. Further, they provide insights into how a stem-restricted memory response impacts the HA-head response elicited following virus challenge.

## Data Availability

The original contributions presented in the study are included in the article/**Supplementary Material**. Further inquiries can be directed to the corresponding author.
